# Multiplex Hydrolysis Probe Real-Time PCR for Simultaneous Detection of Hepatitis A Virus and Hepatitis E Virus

**DOI:** 10.3390/ijms15069780

**Published:** 2014-05-30

**Authors:** Feng Qiu, Jingyuan Cao, Qiudong Su, Yao Yi, Shengli Bi

**Affiliations:** Institute for Viral Disease Control and Prevention, Chinese Center for Disease Control and Prevention, No. 155 Changbai Road, Beijing 102206, China; E-Mails: qiufeng615@sina.com (F.Q.); caojy@126.com (J.C.); 13426329820@163.com (Q.S.); yyao112@163.com (Y.Y.)

**Keywords:** multiplex TaqMan real-time PCR, hepatitis A virus, hepatitis E virus, identification, application

## Abstract

Detection of hepatitis viral infections has traditionally relied on the circulating antibody test using the enzyme-linked immunosorbent assay. However, multiplex real-time PCR has been increasingly used for a variety of viral nucleic acid detections and has proven to be superior to traditional methods. Hepatitis A virus (HAV) and hepatitis E virus (HEV) are the major causes of acute hepatitis worldwide; both HAV and HEV infection are a main public health problem. In the present study, a one-step multiplex reverse transcriptase quantitative polymerase chain reaction assay using hydrolysis probes was developed for simultaneously detecting HAV and HEV. This novel detection system proved specific to the target viruses, to be highly sensitive and to be applicable to clinical sera samples, making it useful for rapid, accurate and feasible identification of HAV and HEV.

## 1. Introduction

Hepatitis A virus (HAV) infection is the leading cause of acute viral hepatitis throughout the world [1]. The virus can survive for extended periods in seawater, fresh water, wastewater and soil. Hepatitis E virus (HEV), the same as HAV, is a small, non-enveloped RNA virus that can also cause acute hepatitis in humans [2]. Both viruses are resistant to freezing, detergents, as well as some chemical reagents, and their route of transmission is usually associated with the consumption of contaminated foods and beverages, such as seafood and pork [3]. The most significant outbreak of HAV infection occurred in Shanghai, China, in 1988, in which almost 300,000 cases were caused by the consumption of clams harvested from a sewage-polluted area [4]. However, numerous outbreaks associated with fruits and raw vegetables have also been reported [5,6]. Similarly, HAV and HEV outbreaks mainly occurred in developing countries where sanitation is not typically available [7].

Because HAV and HEV does not replicate in the environment, water and foods, the traces of viral contamination are difficult to identify [8]. From another perspective, an efficient way to improve the diagnosis of patients suffering from hepatitis is urgently required. Therefore, besides the sensitivity, the accuracy and specificity are also very important for the development of HAV and HEV detection. However, it is very difficult for both HAV and HEV to be grown in cell cultures, which makes the detection by classical culture methods impossible [9]. As a result, molecular techniques offer the best means to determine the viral existence and viral infections, while a hydrolysis probe-based real-time PCR assay provides an effective way for viral nucleic acid detection and amplification [10]. In particular, real-time PCR allows the quantification of a wide range of viral genome copy numbers in different types of samples. Presently, few HAV and HEV single real-time PCR detection systems have been developed [11,12]. However, few reports focus on the simultaneous detection of both HAV and HEV. In this study, we established a novel multiplex real-time PCR assay for the detection of these two hepatitis-associated viruses. It is proven efficient and offers some advantages for the rapid and specific detection of HAV and HEV viral particles. It is expected to become widely used in hepatitis diagnosis and to play important roles in the amplification and determination of HAV and HEV in environmental samples.

## 2. Results

### 2.1. RNA Standard Preparation

**Figure 1:**
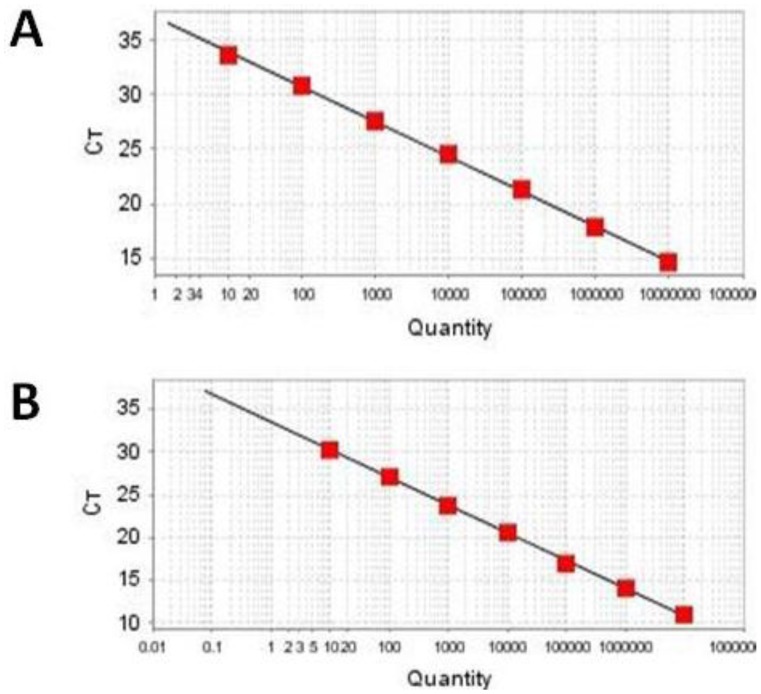
Amplification plots and standard curves for HAV-HEV multiplex TaqMan real-time PCR. The limit of detection and the linearity of the standard curves were determined by using a 10-fold dilution series of HAV and HEV gene fragments ranging from one to 10,000,000 copies. Ct values obtained from the 10-fold dilution series were plotted against the logarithm of the absolute amount of RNA copies. The linear regression equation of the standard curve for hepatitis A virus detection is *Y* = −3.317*X* + 38.228 (*R*^2^ = 0.999) (**A**); The linear regression equation of the standard curve for hepatitis E virus detection is *Y* = −3.923*X* + 45.328 (*R*^2^ = 0.999) (**B**).

We used an HAV RNA fragment synthesized *in vitro* to construct the HAV quantitative standard curve. The RNA molecule was calibrated to 10^7^ copies/μL. Serial dilutions (1:10) of RNA copies with a linear range from 10^7^ to 10^1^ copies were used to achieve a reliable standard curve in the subsequent real-time PCR assay. The linear regression equation of the standard curve is *Y* = −3.317*X* + 38.228 (*R*^2^ = 0.999). The HEV quantitative standard was prepared in the same way as HAV, whereas the linear regression equation of the HEV standard curve is *Y* = −3.923*X* + 45.328 (*R*^2^ = 0.999) (Figure 1).

### 2.2. Precision and Reproducibility Analysis

The repeatability and reproducibility were evaluated by using a 10-fold dilution series of both HAV and HEV gene fragments ranging from 10 to 1,000,000 copies. The real-time PCR assays were performed five times at six different concentrations, and the standard deviation (SD) of the Ct values for repeatability were calculated from the mean values to evaluate the precision of the multiplex real-time PCR. The results are shown in Table 1. These results for the repeatability and reproducibility tests indicate that the multiplex real-time PCR assay was reliable in its detection of HAV and HEV.

**Table 1:** Precision and reproducibility analysis of HAV-HEV multiplex real-time PCR.

Template Quantity (Copies/Reaction)	HAV Detection		HEV Detection	
	Ct Value (Range)	Average	Ct Value (Range)	Average
1,000,000	17.79–18.01	17.89	13.56–13.75	13.71
100,000	21.11–21.36	21.23	16.88–17.08	16.93
10,000	24.58–24.82	24.70	20.19–20.45	20.33
1000	27.88–28.26	28.08	23.51–23.92	23.76
100	31.22–31.60	31.42	26.78–27.32	27.02
10	34.61–35.11	34.83	29.77–30.56	30.35

### 2.3. Sensitivity and Linearity

The limit of detection and the linearity of the standard curves for both HAV and HEV detection systems were determined by using a 10-fold dilution series of HAV and HEV gene fragments ranging from one to 10,000,000 copies. These detections were performed in parallel and in triplicate on the same dilutions. The detection limit was 10 copies for the detection of HAV and HEV, respectively. Ct values obtained from the 10-fold dilution series were plotted against the logarithm of the absolute amount of RNA copies. As a result, a square regression correlation (*R*^2^) of 0.999 and a PCR efficiency of 100% were achieved, representing the good linearity of the multiplex real-time PCR detection system (Figure 1). These results suggest that the detection system was highly sensitive and gave good linearity, as well as high PCR efficiency for targets detection. These results are shown in Table 2.

**Table 2:** Sensitivity analysis of HAV-HEV multiplex real-time PCR.

Template Quantity (Copies/Reaction)	Ct Value (Average Ct for Triplicate Reactions)	
	HAV Template	HEV Template
10,000,000	14.65 ± 0.09	10.28 ± 0.08
1,000,000	17.91±0.14	13.71 ± 0.08
100,000	21.29 ± 0.19	17.05 ± 0.12
10,000	24.78 ± 0.21	20.39 ± 0.18
1000	28.03 ± 0.31	23.73 ± 0.31
100	31.39 ± 0.34	27.12 ± 0.49
10	34.77 ± 0.52	30.54 ± 0.66
1	NA	NA

“NA” represents data beyond the detection limit.

### 2.4. Specificity Analysis

In order to confirm the specificity toward the target viruses of HAV and HEV, a wide variety of enteric viruses and other hepatitis viruses were chosen for amplification by the novel established multiplex real-time PCR. The assessed viruses included enterovirus 71, poliovirus, astrovirus, norovirus GI, norovirus GII, rotavirus, adenovirus 40 and several other hepatitis viruses, such as hepatitis B virus, hepatitis C virus and hepatitis D virus. As expected, none of the tested viruses gave positive results, and no cross-reaction was apparent for any non-target viruses. The results suggest that the designed primer pairs and probes were highly specific and selective for their target viruses and exhibited no cross-reactivity with other species.

### 2.5. Application of Multiplex Real-Time PCR to Clinical Sera Samples

The applicability of the multiplex real-time PCR was investigated by the analysis of the sera samples from acute hepatitis patients. A panel of fifty blood serum samples collected from patients with HAV infection and another panel of fifty blood serum samples with HEV infection were employed. After multiplex real-time PCR amplification, 47 HAV sera samples gave positive results, whereas 45 of the HEV samples tested positive. This was approximately consistent with the clinical diagnosis by enzyme-linked immunosorbent assay. Furthermore, we calculated viral gene copies in 1 mL of each serum sample by quantification with the RNA standard curve. The virus copies obtained from HAV sera samples ranged from 5.18 × 10^2^ to 4.93 × 10^7^/mL, whereas virus copies obtained from HEV sera samples ranged from 9.33 × 10^3^ to 4.06 × 10^7^/mL.

## 3. Discussion

Both HAV and HEV infection are mainly propagated via the fecal-oral route, which is highly associated with a lack of sanitation infrastructure. The distribution patterns of hepatitis A and hepatitis E in different geographical areas of the world are closely related to socioeconomic development [13]. The endemicity is low in developed regions and high in underdeveloped countries. China, as the largest developing country in the world, urgently need an efficient way for the rapid detection of HAV and HEV infection and hepatitis clinical diagnosis.

Nowadays, real-time PCR has become an essential diagnostic technique. The main advantage of this technique is that with real-time PCR, the starting template copy number can be determined with accuracy and high sensitivity over a wide dynamic range. It can even detect a single copy of target gene. In addition, real-time PCR assays can reliably detect gene copies with lower coefficients of variation than other detection methods. Another advantage of real-time PCR is that data can be evaluated without gel electrophoresis, resulting in reduced experiment time. Finally, the real-time PCR assay is performed and data are evaluated in a closed-tube system, eliminating the need for post-amplification manipulation and, thus, reducing opportunities for contamination. The detection of the target via binding of a probe, such as hydrolysis probes, is more specific than that via binding of SYBR green, especially for RNA viruses that have highly variable genomes. The simultaneous detection method for HAV and HEV, described in this study, is a multiplex real-time PCR based on a hydrolysis probe technique. It is an improvement and upgrade of the uniplex real-time PCR for single virus detection. Therefore, it is more convenient than and superior to HAV and HEV uniplex real-time PCR.

First of all, the development of an optimal multiplex real-time PCR methodology is mainly based on the proper selection of the target sequences for primers and probe annealing. The choice of appropriate primer and probe binding sites is very crucial. The selected targets must guarantee an absolute specificity and must reach equilibrium between the high sensitivity, broad reactivity and reliability of quantification. For HAV RNA detection and quantification, most procedures chose the highly preserved 5'-UTR region as an amplification target, whereas ORF2 in the HEV genome was used extensively for HEV RNA quantification [11,12,14,15]. In our study, we chose the highly conserved region in the 5'-UTR in the HAV genome and ORF2 in the HEV genome for designing the primer pairs and probe targets. Our design proved very effective in the subsequent amplification experiments. The multiplex real-time PCR assay established in the current study is effective for identifying HAV and HEV independently in one run, allowing for rapid detection, reduced costs and less sample waste when compared to similar assays using uniplex PCR [11,12,14,15]. Besides superior stability and reproducibility (Table 1), the detection limit of the multiplex real-time PCR was 10 copies per reaction for detection of HAV and HEV, respectively, suggesting that it has the same sensitivity as HAV/HEV uniplex real-time PCR [11,12]. Compared with a similar study by other researchers [16], the duplex real-time PCR developed in this study was a little less sensitive than the simultaneous quantification system of hepatitis virus A, B, C and E established by Irshad M. *et al*., the detection limits of which were 280  copies/mL for HAV and 300  copies/mL for HEV. For our interest, for a similar concentration of HAV and HEV templates, multiplex real-time PCR gave very different Ct values (Table 1 and Table 2), indicating the different PCR efficiency of HAV and HEV templates during amplification. On the other hand, the specificity of the proposed primer-probe sequences has been shown to be excellent against a broad panel of enteric viruses and other hepatitis viruses, including hepatitis B virus and hepatitis C virus. Moreover, the applicability of the multiplex real-time PCR was validated by the examination of the sera from acute clinical hepatitis patients. As expected, viral RNA detection was significantly correlated with the detection by the traditional method of enzyme-linked immunosorbent assay, although there were 3/50 HAV-IgM positive samples and 5/50 HEV-IgM positive samples proven negative in nucleic acid detection. These inconsistent results could be ascribed to the following reasons. First of all, IgM can reappear in serological tests in up to a third of infected people during recurrences, and the unexplained persistence of IgM years after the primary infection also existed. Therefore, IgM tests can lead to deceptive test results. Secondly, IgM tests sometimes cross-react with other viruses in the same family, such as enterovirus 71, which causes hand-foot-and-mouth disease, meaning that positive results may be misleading. Finally, there are other factors, such as the rheumatoid factor, which could also disturb the IgM assay.

## 4. Methods

### 4.1. Ethics Issues

All aspects of the study were performed in accordance with the national ethics regulations and approved by the ethics committee of the Chinese Center for Disease Control and Prevention.

### 4.2. Clinical Specimens from Patients with Acute HAV Infection and HEV Infection

A panel of fifty blood serum samples collected from patients with confirmed acute HAV infection and another panel of fifty blood serum samples with HEV infection were employed to evaluate the clinical application of the multiplex real-time PCR assay. These HAV and HEV cases were confirmed on the basis of a positive result in the enzyme-linked immunosorbent assay (Kehua Bio-engineering, Shanghai, China) for HAV and HEV IgM antibody tests. All serum samples were stored at −20 °C until further use.

### 4.3. Viral RNA Extracted from Clinical Sera Specimens

Genomic viral RNA was extracted from 140 µL of serum samples using a QIAamp viral RNA mini kit (Qiagen, Hilden, Germany), according to the manufacturer’s instructions. RNA was eluted from the QIAamp spin column in a final volume of 50 µL with RNase-free water and stored at −80 °C until use.

### 4.4. RNA Standard Preparation

According to our previous study [17], two plasmids containing the HAV genome and HEV genome were used as the *in vitro* transcriptional templates, respectively. The transcription products, HAV and HEV fragments, were treated with RQ1 DNase (Promega, Madison, WI, USA) (1 U/µg cDNA) to remove the DNA template and purification using an RNeasy^®^ Mini Kit (Qiagen, Hilden, Germany), according to the manufacturer’s instructions. The quality and the concentration of our prepared RNA was measured using NanoDrop™ equipment (ThermoFisher, Wilmington, DE, USA). RNA integrity and purity could be assessed by the ratio of the optical density values absorbed at 260 and 280 nm. A value of 1.8–2.1 indicated that the RNAs were pure. In addition, the weight of RNA fragments could be estimated at the same time, followed by the calculation of RNA copies in the solution. After the transcription, purification and RNA quality evaluation, 10-fold series dilutions of HAV and HEV gene fragments were performed to prepare the RNA quantification standard ranging from 1 to 10,000,000 copies.

### 4.5. Primer and Probe Design for HAV-HEV Multiplex Real-Time PCR

The development of an optimal real-time RT-PCR methodology relies on the proper selection of the target sequences for primers and probe annealing. In this study, HAV and HEV sequences available in GenBank (HAV sequence accession numbers: X75215, M14707, AF357222, AF512536, AB020568, AB020569, AB258583, AB253604, AY644676, AY644670, D00924; HEV sequence accession numbers: M73218, D10330, X99441, M80581, M94177, D11092, AF076239, AF455784, M74506, AF082843, AF060668, AB089824, AB073912, AY115488, AB097812, AB091394, AB099347, AB108537) were analyzed, and the primer-probe set was designed on the basis of highly conserved regions of the HAV and HEV genome. The primer and probe sequences, fluorophores and quenchers used are shown in Table 3. The positions were numbered according to the complete sequence of the HAV HM175 strain (GenBank accession number: M14707) and HEV Burma strain (Genebank accession number: M73218).

**Table 3:** Primers and hydrolysis probes used in the experiment.

Targeted Viruses	Name	Sequence of Oligonucleotide (5'–3')	Location	Amplicon Length
Hepatitis A Virus	Forward primer	GGT AGG CTA CGG GTG AAA C	393~411 ^a^	116 nt
	Reverse primer	CCT CCG GCG TTG AAT GGT TT	489~508 ^a^	
	Probe	FAM-ACA GCG GCG GAT ATT GGT GAG TTG TTA AGA-BHQ	456~485 ^a^	
Hepatitis E Virus	Forward primer	GGT GGT TTC TGG GGT GAC	5261~5278 ^b^	70 nt
	Reverse primer	AGG GGT TGG TTG GAT GAA	5313~5330 ^b^	
	Probe	Cy5-TGA TTC TCA GCC CTT CGC-BHQ	5284~5301 ^b^	

FAM, fluorescein amidite; BHQ, black hole quencher; Cy5, cyanine 5; ^a^ corresponding nucleotide position of HAV virus (HM175) (Accession No. M14707); ^b^ corresponding nucleotide position of HEV virus (Burma) (Accession No. M73218).

### 4.6. Multiplex Real-Time PCR Conditions

The multiplex real-time PCR assays were carried out in a 96-well format using the One Step PrimeScript™ RT-PCR Kit (Takara, Dalian, China). Briefly, 5 µL of extracted viral RNA was transferred into a capillary containing 20 µL master mix containing a final concentration of 200 nM of each primer and 100 nM of each probe. RT-PCR was performed at the optimized condition, including 3 steps. Reverse transcription was performed at 42 °C for 30 min followed by denaturation at 95 °C for 10 s. Amplification was achieved by 40 cycles of 95 °C for 8 s and 60 °C for 34 s. All reactions were carried out on an ABI 7500 real-time RT-PCR system (Applied Biosystems, Foster City, CA, USA).

The results were analyzed using ABI 7500 software (Applied Biosystems). For each amplification plot, the Ct value, representing the PCR cycles at which the reporter dye fluorescence was detectable above the fluorescence threshold, was calculated automatically. The use of two fluorophores allowed the differentiation of the virus targets.

## 5. Conclusions

It is concluded that the multiplex real-time RT-PCR assay developed in this work is a highly sensitive and specific tool for the accurate quantification of hepatitis A virus and hepatitis E virus simultaneously. Since it was validated by testing clinical sera specimens from acute hepatitis patients, further research is planned to investigate its effectiveness for detecting HAV and HEV in environmental samples, such as waste water and polluted fruits. We anticipate that multiplex real-time RT-PCR will be a useful, rapid and advanced tool for monitoring HAV and HEV in clinical samples, as well as environmental samples during routine and epidemic investigations.
